# High-Density Lipoprotein Function and Dysfunction in Health and Disease

**DOI:** 10.1007/s10557-018-06846-w

**Published:** 2019-01-24

**Authors:** Scott T. Chiesa, Marietta Charakida

**Affiliations:** 1 Vascular Physiology Unit, UCL Institute of Cardiovascular Science, 1 St. Martin’s Le Grand, London, EC1A 4NP UK; 2 Division of Imaging Sciences and Biomedical Engineering, King’s College London, London, UK

**Keywords:** HDL dysfunction, Inflammation, Atherosclerosis, Cardiovascular disease

## Abstract

High-density lipoprotein cholesterol (HDL-c) has long been referred to as ‘good cholesterol’ due to its apparent inverse relationship with future CVD risk. More recent research has questioned a causal role for HDL-c in this relationship, however, as both genetic studies and numerous large-scale randomised controlled trials have found no evidence of a cardiovascular protective effect when HDL-c levels are raised. Instead, focus has switched to the functional properties of the HDL particle. Evidence suggests that both the composition and function of HDL may be significantly altered in the context of an inflammatory milieu, transforming the particle from a vasoprotective anti-atherogenic particle to a noxious pro-atherogenic equivalent. This review will summarise evidence relating HDL to CVD risk, explore recent evidence characterising changes in the composition and function of HDL that may occur in chronic inflammatory diseases, and discuss the potential for future HDL-modifying therapeutic interventions.

## Introduction

High-density lipoproteins (HDL) participate in the transfer of excess cholesterol from peripheral sites to the liver, and the cholesterol carried within these lipoproteins (HDL-c) is therefore often referred to as ‘good cholesterol’. Over the past half-century, HDL-c has repeatedly been shown to be inversely associated with risk of future cardiovascular events, and has therefore become one of the most well-known and widely studied risk factors for cardiovascular disease (CVD). Recent research, however, has called the causal nature of this relationship into question, with genetic studies finding little evidence of an association between elevated HDL-c levels and reduced CVD risk [1, 2], and a series of major clinical trials failing to demonstrate any clinical benefit when HDL-c levels were pharmacologically raised [3–7]. Recent insights into the complex structural and functional properties of HDL particles—and their vulnerability to modification when exposed to a range of CV risk factors, particularly inflammation—have shed light on the potential reasons for these negative findings, and have posed the question as to whether focusing on the ‘quality’ rather than quantity of HDL may be a more relevant target for drug development. This review will summarise previous research attempting to reduce CVD risk through the modification of HDL-c levels, explore recent evidence characterising changes in the composition and function of HDL that may occur in chronic inflammatory diseases, and discuss the potential for future HDL-modifying therapeutic interventions.

## HDL-C and Cardiovascular Risk: When Correlation Does Not Equal Causation

The concept of HDL-c as a beneficial lipid was first proposed in the Framingham Heart Study in the latter half of the twentieth century, where low levels of HDL-c were found to be an independent risk factor for future incidence of coronary heart disease (CHD) in 2815 men and women over 12 years of follow-up [8, 9]. The replication of these findings in further large-scale cohorts suggested a protective role for HDL-c in the atherosclerotic process [10, 11], leading to the adoption of HDL-c’s well-known description as a form of“good cholesterol”. Despite robust relationships in epidemiological studies, however, the observational nature of these findings long meant that the question of whether HDL-c played a direct role in the disease process—or was simply a biomarker of other underlying complications—remained uncertain.

Disappointingly, a number of findings from genetic studies and numerous large-scale randomised clinical trials in the last 10–15 years have suggested the latter. Firstly, the development of genome-wide association studies (GWAS) and Mendelian Randomization studies in the early 2000s allowed the causal nature of this relationship to be investigated for the first time using HDL-raising genetic variants in large observational studies as proxy measures for cumulative HDL-c exposure. Findings from two separate Mendelian Randomization studies containing over 165,000 participants found no association between genetically determined HDL-c and risk of myocardial infarction, despite traditional observational relationships from the same cohorts displaying the expected inverse relationship with plasma levels of HDL-c [1, 2]. These findings challenged the concept that elevating HDL-c would translate into clinical benefit, and these concerns were later confirmed by negative findings from numerous randomised clinical trials investigating a number of different HDL-raising drugs. Perhaps the most well-known of these failures was that of the ILLUMINATE trial, a randomised clinical trial investigating the use of torcetrapib in>15,000 individuals at high risk of CVD, and the first major trial involving a recently developed class of drugs known as cholesterol ester transfer protein (CETP)-inhibitors. As the name suggests, this drug class—which included the agents torcetrapib, dalcetrapib, evacetrapib, anacetrapib, and obicetrapib—was designed to block CETP; the enzyme responsible for transferring cholesterol esters away from HDL and on to VLDL/LDL. Despite raising HDL-c levels by over 70%, ILLUMINATE was forced to terminate prematurely due to an increased risk of CV events and mortality in patients taking the drug, most likely due to off-target effects on aldosterone production and blood pressure [3]. The development of different forms of this drug that lacked these off-target effects maintained hope for CETP-inhibitors as a therapeutic target, but results from later trials proved once again disappointing, with both the dal-OUTCOMES (dalcetrapib) and ACCELERATE (evacetrapib) trials showing no benefit of treatment despite significant increases in HDL-c [4, 5]. Although a rare positive outcome was reported for anacetrapib—the final CETP-inhibitor to be tested in the recent HPS3/TIMI55-REVEAL trial—this was concluded to have likely resulted from a further lowering of LDL-c rather than the observed elevation in HDL-c [12]. Alongside CETP-inhibitors, the early 2000s also saw the design and completion of a number of trials investigating extended release niacin, an agent shown to consistently elevate HDL-c via numerous distinct pathways—including (but not limited to) an upregulation of ApoA-I production, a reduction in CETP activity, and a reduction in hepatic uptake of HDL [13]. In the first of these studies (AIM-HIGH), the addition of niacin to statin treatment in >3400 patients showed no evidence of clinical benefit, leading the trial to be terminated early due to lack of efficacy [6], whereas in the later HPS2-THRIVE trial, extended release niacin not only had no clinical benefit, but was in fact found to increase risk of serious adverse events [7]. Taken together, findings from these trials appear to have ended the concept that elevated levels of HDL-c act as a protective factor against atherosclerotic disease, and efforts to reduce CVD risk through pharmacological manipulation of HDL-c are therefore no longer being pursued.

## HDL Function and Cardiovascular Risk: a Shift of Focus from ‘Quantity’ to ‘Quality’

Given the wide range of anti-atherogenic properties attributed to the HDL particle in decades of animal research and preclinical studies, the discovery that HDL-c lacked a causal role in CVD risk was initially unexpected. However, the majority of functional properties exerted by HDL are not dependent on the cholesterol content of the particle per se, but rather on numerous other structural components contained within the complex and highly heterogenous HDL particle. Therefore, with the end of the HDL-c hypothesis in the early 2000s came the start of the HDL function hypothesis—characterised by a focus on the functional capacity of HDL particles, rather than their absolute cholesterol levels alone.

**Fig. 1:**
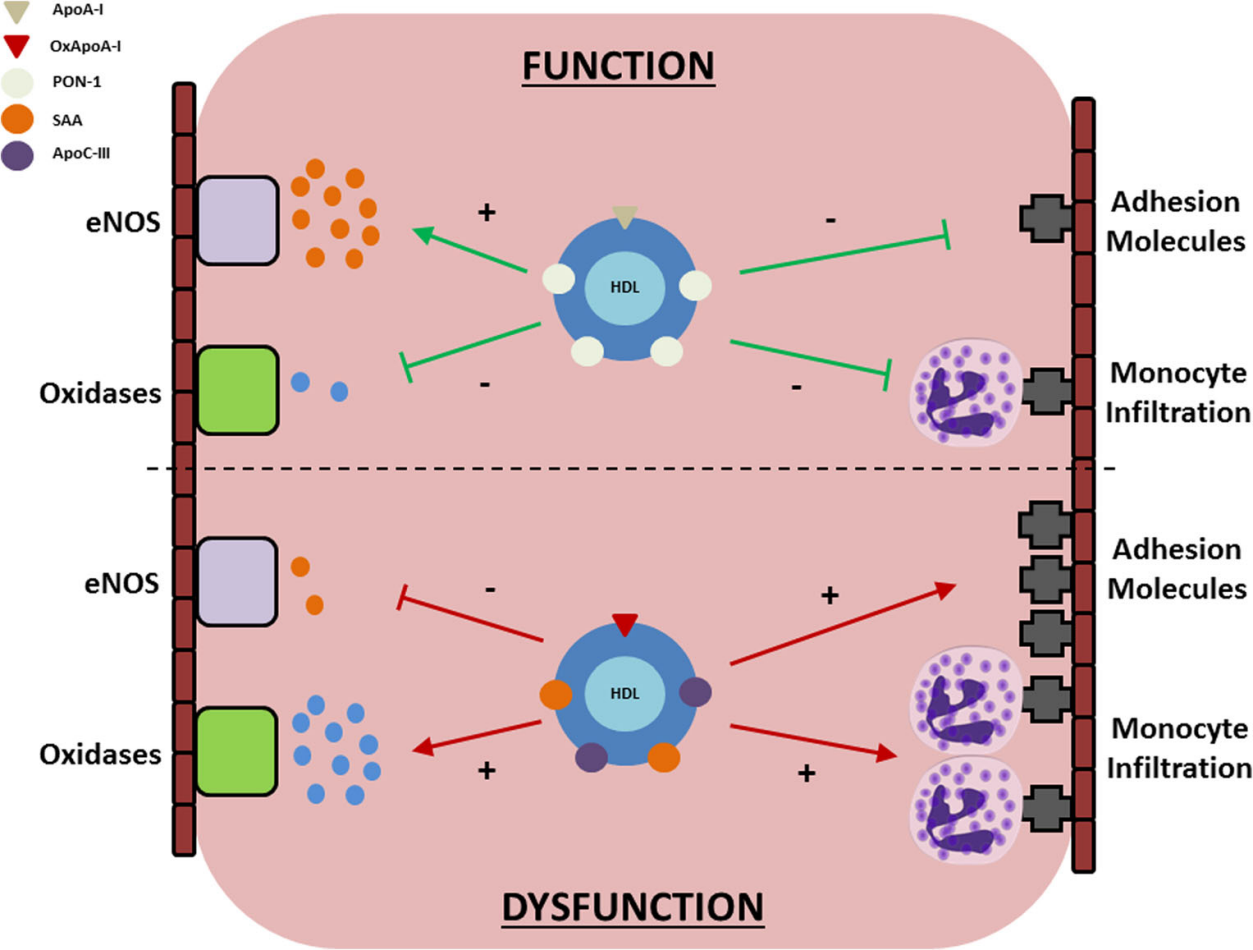
Potential mechanisms linking dysfunctional HDL to vascular damage. In the absence of inflammation (top), HDL exerts vascular protective effects through its ability to increase endothelial NO bioavailability, decrease oxidative stress, and inhibit the expression of adhesion molecules on the vascular wall. In inflammatory states (bottom), however, numerous structural and functional changes may occur and compromise endothelial function. Beneficial proteins contained within the particle (e.g. ApoA-I, PON-1) are liable for oxidation or replacement, resulting in a noxious particle which impairs NO generation, amplifies oxidative stress, and promotes adhesion molecule expression and monocyte infiltration across the vascular wall

In the basal state, HDL’s functional properties are predominantly anti-atherogenic; the most well-studied of which is its ability to facilitate the removal of excess cholesterol from atherosclerotic plaques in a process known as reverse cholesterol transport (RCT). In addition to RCT, however, HDL has also been shown to have multiple other vascular effects which may be expected to provide additional protection; such as an ability to increase endothelial nitric oxide (NO) bioavailability, a capacity to reduce oxidative stress and inflammation, and an ability to reduce the expression of endothelial adhesion markers and transendothelial monocyte migration [14]. More recent evidence, however, has highlighted the presence of numerous structural and functional changes in the HDL particle that may result in its transformation from an anti-atherogenic vasoprotective particle to a pro-inflammatory noxious equivalent (Fig. 1). These alterations are most commonly observed in the presence of systemic inflammation, and suggest an evolutionary role for HDL in the innate immune response, where the greatest threat to human longevity has long come from viral or bacterial infections [15]. With long-term chronic inflammatory conditions such as CHD or type 2 diabetes (T2D) now prevalent in the population, however, the possibility exists that this previously ‘protective’ response may now paradoxically accelerate vascular damage and increase the risk of mortality via atherosclerotic complications.

The remainder of this review will therefore seek to address this topic, beginning with (1) evidence surrounding the anti-atherogenic role for HDL, (2) changes in its structure and function that may accompany chronic inflammation, (3) the implications these changes have for CVD risk in different clinical populations and (4) what this may mean for HDL as a risk marker or therapeutic target.

## HDL Structure and Function in Healthy Individuals: an Anti-atherogenic Particle Exerting Multiple Vasoprotective Effects

### HDL Structure

The term HDL in fact refers to a highly heterogeneous range of particles within human plasma, ranging in size from 5 to 17 nm and density from 1.063–1.210 kg/l. Alongside cholesterol, lipidomic and proteomic techniques have identified over 200 additional lipids and more than 85 proteins which may reside within the HDL particle [16, 17], as well as multiple enzymes and even genetic material in the form of micro RNAs (Table 1). Significant variation in the relative quantities of these components results in a wide spectrum of circulating HDL particles that may differ in size, density, structure, and function. For ease of assessment, however, these are commonly separated into five broad subclasses, ranging from the largest and least dense (termed HDL_2b_) to the smallest and most dense (termed HDL_3b_). The major structural protein component of HDL is apolipoprotein A-I (apoA-I), formed in both the liver (~80%) and intestine (~20%) and secreted in a lipid-free state. Efflux of phospholipids and free cholesterol on to at least 2 molecules of apoA-I stimulate the biogenesis of HDL, resulting in the generation of a discoidal lipid-poor ‘nascent’ HDL particle. Subsequent incorporation of numerous enzymes such as lecithin-cholesterol acyltransferase (LCAT), cholesterol ester transfer protein (CETP) and phospholipid transfer protein (PLTP) into this nascent particle further alter the lipid and phospholipid composition—resulting in the generation of mature spherical particles of differing sizes and densities. These particles may be further modified through the incorporation of numerous other structural proteins and enzymes (shown in Table 1) all of which interact to determine the eventual functional capabilities of the HDL particle [18].

**Table 1:** Structural components contributing to heterogeneity of HDL molecule

Shape	Subclasses (size/density)	Apolipoproteins	Lipids	Enzymes	Acute phase proteins	miRNAs
Discoidal (Nascent)	HDL_2b_ (Largest / Least Dense)	ApoA (I, II, IV, V)	Cholesterol	LCAT	SAA	miR-92a
Spherical (Mature)	HDL_2a_	ApoC (I, II, III, IV)	Cholesterol esters	PLTP	LBP	miR-126
	HDL_3a_	ApoD	Triglycerides	CETP	Fibrinogen	miR-150
	HDL_3b_	ApoE	Glycerophosphlipids	Lp-PLA_2_	C3	miR-223
	HDL_3b_ (Smallest / Most Dense)	ApoF	Sphingolipids	MPO	A1AT	
		ApoH		PON-1		
		ApoJ				
		ApoM				

*HDL*, high-density lipoprotein; *Apo*, apolipoprotein; *LCAT*, lecithin cholesterol acyl-transferase; *PLTP*, phospholipid transfer protein; *CETP*, cholesterol ester transfer protein; *Lp-PLA*_*2*_, lipoprotein-associated phospholipid A2; *MPO*, myeloperoxidase; *PON*, paraoxonase; *SAA*, serum amyloid A; *LBP*, lipopolysaccharide binding protein; *C3*, complement C3; *A1AT*, alpha-1-anti-trypsin

### Reverse Cholesterol Transport

**Fig. 2:**
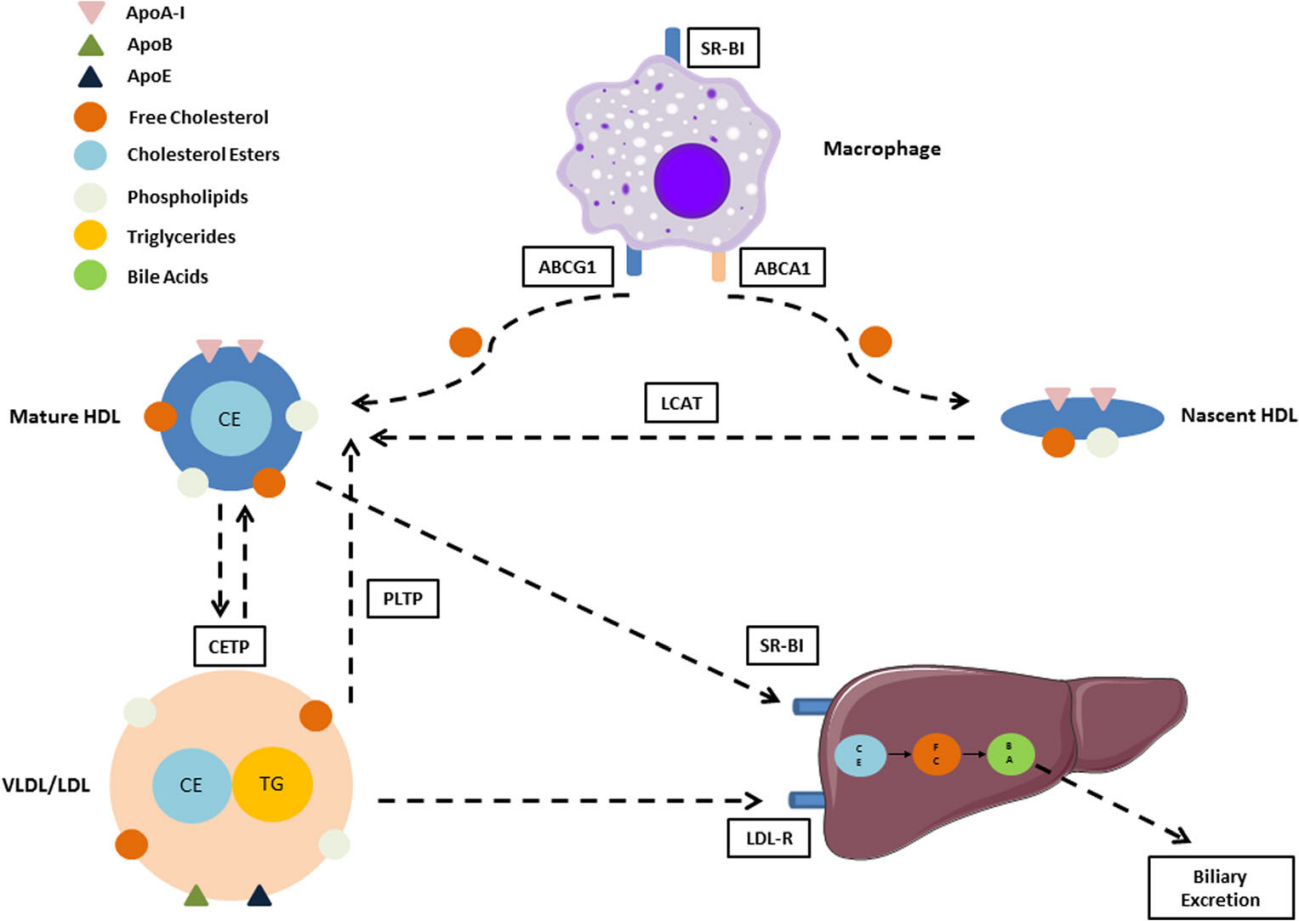
Mechanistic pathways underlying reverse cholesterol transport from macrophages to liver. HDL-mediated RCT involves the removal of cholesterol from lipid-laden macrophages, where it may then be transported back to the liver for biliary excretion. In healthy individuals, nascent and mature HDL particles accept cholesterol from lipid-laden macrophages within the vascular wall via an interaction of ApoA-I with ABCA1 and ABCG1 receptors. Following their esterification by the enzyme LCAT, these cholesterol particles may undergo selective uptake to the liver via the interaction of HDL and hepatic SR-BI, or may be transferred by CETP to VLDL/LDL for hepatic uptake via LDL-R. In conditions of chronic disease or inflammation, numerous changes in this pathway have been observed that may render it dysfunctional and potentially increase atherosclerotic risk. These changes include the oxidation or displacement of ApoA-I, alterations in the activity of enzymes such as LCAT or CETP, and reduced binding of HDL to receptors such as ABCG1 or SR-BI

The concept of HDL as a shuttle for removing excess cholesterol from lipid-laden macrophages within peripheral tissues was first proposed over 50 years ago [19], and since then has become one of the most well-recognised pathways underlying HDL’s anti-atherogenic nature (Fig. 2). When cell cholesterol content is normal, this process is dominated via the passive aqueous diffusion of free cholesterol between cell membranes and HDL [20]. Although this pathway is bidirectional, esterification of free cholesterol within HDL by the enzyme LCAT maintains a concentration gradient promoting efflux out of the cells, thereby preventing its accumulation in cell membranes and maintaining homeostasis. In addition to passive diffusion, non-aqueous efflux may also occur through the binding of HDL to scavenger receptor class B1 (SR-BI), with this pathway shown to be enhanced when absolute HDL levels are low, or HDL particle size is large [21]. When cell cholesterol content is elevated, however—as is the case in atherogenic lipid-laden macrophages—a shift to active efflux pathways mediated through the upregulation of the ATP-binding cassette transporters ABCA1 and ABCG1 begin to predominate. The most important of these pathways involves the efflux of phospholipids and free cholesterol on to lipid-free or lipid-poor apolipoproteins via ABCA1, as demonstrated by the inability of ABCA1-knockout mice [20] or patients with Tangier’s disease (who lack ABCA1) to generate HDL [22]. Although all HDL-associated apolipoproteins are capable of accepting cholesterol from ABCA1, interactions with ApoA-I account for the majority of the response [23]. As is the case for passive diffusion, LCAT-mediated esterification of cholesterol on these newly formed nascent HDL particles reduces free extracellular free cholesterol levels by trapping them as esters within the HDL core; with this process both maintaining the concentration gradient and resulting in the development of mature spherical HDL particles capable of mediating further efflux through ABCG1 receptors [24]. Once transferred to HDL, cholesterol esters are then returned to the liver for biliary excretion in one of two ways—they may be exchanged for triglycerides in VLDLs and other remnant lipoproteins by CETP, thereby enabling their uptake and excretion via hepatic LDL receptors, or they may be selectively removed from HDL directly via its binding to hepatic SR-BI [25].

### Endothelial Nitric Oxide Bioavailability

Diminished nitric oxide (NO) bioavailability is a hallmark of the vascular disease process, and is thought to underpin every stage of atherosclerosis [26]. The first evidence of HDL’s ability to directly promote endothelial NO production came in the early 2000s, where the addition of HDL to cultured endothelial cells was shown to directly stimulate endothelial NO synthase (eNOS) activation in vitro [27]. In this ground-breaking study, the authors demonstrated an essential role for both ApoA-I and SR-BI in NO generation, as removal of the former using antibodies, or the latter using SR-BI knockout mice, resulted in a loss of effect. However, the use of purified ApoA-I in place of HDL also failed to elicit a response, suggesting a necessary involvement of other components within the HDL particle for eNOS phosphorylation. Later research identified a number of HDL-bound bioactive phospholipids—namely sphingosylphosphorylcholine (SPC), sphingosine-1-phosphate (S1P), and lysosulfatide (LSF)—as at least partially responsible [28], with the binding of these to sphingosine-1-phosphate receptor 3 (S1P3) demonstrated to stimulate vasorelaxation via the activation of phosphatidylinositol 3-kinase(PI3K)/Akt and mitogen-activated protein kinase (MAPK) pathways [29]. In addition to ApoA-I and SR-BI, additional ABCG1 pathways are also likely contribute to this response. HDL-mediated efflux of 7-ketosterol via ABCG1 has also been shown to improve NO release by preventing the generation of NO-scavenging reactive oxygen species within the endothelium, while cholesterol efflux via the same receptor has been shown to elicit similar responses via a reduction in eNOS/caveolin interactions [30, 31].

### Anti-oxidant and Anti-inflammatory Capacity

An accumulation of foam cells within the vascular wall is another key process of atherosclerotic disease, initiated via the uptake of circulating LDL-c and its subsequent oxidation by reactive oxygen species (ROS) and pro-inflammatory enzymes such as MPO within the sub-endothelial space. This oxLDL is subsequently taken up by scavenger receptors on macrophages, triggering a cascade of further oxidative and inflammatory responses which encourage the promotion of endothelial adhesion molecule expression and increased monocyte infiltration within the vessel wall. HDL has been shown to exert multiple antioxidant and anti-inflammatory effects that may reduce this vicious circle of oxidation/monocyte infiltration, such as an ability to protect endothelial and smooth muscle cells from the cytotoxic effects of oxLDL [32–34] in vitro*.* This effect is likely to be attributed to a variety of mechanisms. For example, increases in NO bioavailability (as described in the previous section) likely contribute at least in part to this response, as do the presence of multiple antioxidant enzymes carried within HDL’s protein cargo. The most studied of these enzymes is the antioxidant enzyme paraoxonase-1 (PON-1), although roles for other enzymes such as lipoprotein-associated phospholipid A2 (Lp-PLA_2_) [35] and LCAT [36] have also been demonstrated. The presence of PON-1 has been shown to protect both HDL and LDL from oxidation in vitro [33, 37], while its absence (using PON-1 knockout mice) has been demonstrated to have the opposite effect [38]. Interactions with ApoA-I appear to be crucial for its activity, as demonstrated by the significantly increased capacity for PON-1 to prevent LDL oxidation and promote RCT in HDL particles containing ApoA-I as opposed to those containing ApoA-II or IV [39]. Additional antioxidant effects of ApoA-I also likely contribute to HDL’s antioxidant properties via its ability to directly bind and remove oxidised lipids from LDL particles within the vascular wall, as treatment of arterial cell walls with ApoA-I or an Apo-AI mimetic peptide in vitro prevents the oxidation of LDL, as does injection of ApoA-I into both mice and humans [40, 41]. HDL has also been shown in a number of studies to reduce superoxide production in endothelial cells treated with tumour necrosis factor alpha (TNF-α) [42–44], possibly through inhibitory effects on nicotinamide adenine dinucleotide phosphate (NADPH)-oxidases mediated through HDL-associated lysosphingolipids and their interaction with S1P3 and SR-BI receptors [45]. Both this pathway and others have also been shown to have downstream effects on the production of numerous inflammatory-mediated adhesion molecules such as vascular and intercellular adhesion molecules (VCAM-1 and ICAM-1) [46], E-selectin [28], and monocyte chemoattractant protein-1 (MCP-1) [45, 47], reducing their expression and limiting monocyte transmigration across the vascular wall. Furthermore, ABCA1-mediated cholesterol efflux to ApoA-I may also provide additional suppression through the activation of anti-inflammatory signalling molecules during reverse cholesterol transport [48].

## HDL Structure and Dysfunction in Chronic Inflammation: When “Good Cholesterol” Turns Bad

### Inflammation Alters HDL Structure

The concept that individuals with chronic disease may have structurally modified and potentially dysfunctional HDL was initially suggested in the mid-1990s, where evidence was produced for the first time showing the replacement of ApoA-I and paraoxonase-1 (PON-1) during an acute inflammatory response with acute phase proteins such as ceruloplasmin and serum-amyloid A (SAA) [47]. In this seminal study, the authors further noted that the antioxidant and anti-inflammatory vasoprotective properties of these modified HDL particles were also lost—or in certain cases—even completely reversed, suggesting that conformational changes in the HDL particle may have negatively affected its function. Since then, wide-ranging structural changes have been reported in a variety of inflammatory disease states, many of which have been implicated in the generation of a dysfunctional phenotype which may act to increase atherosclerotic risk. The most well-studied of these is the incorporation of acute phase proteins such as SAA, symmetric dimethylarginine (SDMA), lipopolysaccharide binding protein (LBP), alpha-1-antitrypsin (A1AT), or fibrinogen into HDL’s protein cargo [49]. These changes in turn result in reciprocal and detrimental reductions in ApoA-I, a decrease in the activity of HDL-associated antioxidant enzymes such as PON-1 and Lp-PLA_2_, and an increased presence of inflammatory enzymes and lipid peroxidation products such as myeloperoxidase (MPO) and malondialdehyde (MDA) [49]. Furthermore, compositional changes in HDL’s lipid cargo—such as the enrichment with triglycerides commonly observed in hypertriglyceridaemic states—may further affect particle size and density, and therefore functional ability.

### Inflammation Reduces Reverse Cholesterol Transport (RCT)

While a reduction in cholesterol efflux is a well-established aspect of the innate immune system during acute infections, it may have long-term negative implications under conditions of chronic inflammatory stress [15]. Numerous studies have shown that this vital homeostatic process may become impaired by a number of structural and conformational changes within HDL particles exposed to acute or chronic inflammatory conditions. The incorporation of SAA has been shown to impair cholesterol efflux in some [50–53], but not all [42, 54], studies. These changes may arise through the displacement of atheroprotective components such as ApoA-I or PON-1 within the HDL particle itself [50], or by interactions with cell membrane-bound receptors responsible for binding HDL during the RCT process. In mice overexpressing SAA, reduced binding of HDL to SR-BI and a reduction in selective hepatic cholesterol ester uptake from HDL has been observed in conjunction with elevated levels of lipid-free SAA, suggesting a reduced capacity for the liver to remove cholesterol esters for excretion [53]. Further upstream in the RCT pathway, genetic ablation of SAA has been observed to preserve cholesterol efflux in mice following an endotoxin challenge, whereas the same challenge in humans resulted in progressive decreases in efflux capacity in line with elevations in the content of HDL-incorporated SAA [51]. Furthermore, an increased affinity for HDL to bind to proteoglycans at peripheral sites has also been demonstrated following SAA elevation in endotoxin-injected mice [55]. This effect is again virtually abolished following SAA knockout, suggesting that the incorporation of SAA into HDL may further impair RCT through the entrapment of HDL within vascular lesions, where its contents may subsequently be liable to oxidation or enzymatic modification that render it more atherogenic [55]. In support of this, a number of studies have demonstrated extensive structural and functional changes within HDL particles located within the vessel wall in comparison to those circulating in the bloodstream [56–58], resulting in a substantially dysfunctional HDL phenotype which is unable to promote cholesterol efflux. For example, ApoA-I isolated from human atherosclerotic plaques has been shown to be lipid-poor, heavily oxidised by the inflammatory enzyme myeloperoxidase (MPO), and virtually devoid of any cholesterol acceptor activity [59]. This dysfunctional form of ApoA-I—which may also be caused by chlorination, nitration, or sulfoxidation of other amino acid residues—appears to exert its detrimental effects via multiple sites of action, including impairment of ABCA1 cholesterol efflux [58, 60, 61] and decreased ApoA-I-induced activation of LCAT [62]. Further inhibition of ABCA1 via an accumulation of the lipid peroxidation product MDA (a reactive carbonyl that has been shown to increase in HDL in chronic disease states) has also been demonstrated [63], while HDL-enrichment with triglycerides has been shown to impact RCT in some, but not all, studies. These latter findings may perhaps seem counter-intuitive given the finding that triglyceride-enriched HDL may in fact increase macrophage cholesterol efflux [64–66]. However, they are likely explained by further downstream effects that may affect overall RCT, including a reduced ability for LCAT to convert free cholesterol to cholesterol esters [64], and a diminished capacity to deliver these cholesterol esters to hepatic cells via SR-BI [64, 67]. HDL remodelling due to elevated PLTP activity may also adversely affect cholesterol acceptor ability, and has been shown to result in increased atherosclerotic plaque formation in mice [68].

### Inflammation Reduces Endothelial Nitric Oxide Bioavailability

The ability of HDL to promote endothelial nitric oxide bioavailability has repeatedly been shown to be impaired in multiple chronic inflammatory and chronic disease states [42–44, 69–71]. HDL isolated from patients with either established coronary heart disease or acute coronary syndrome has a reduced capacity for NO generation that appears mediated, at least in part, by activation of endothelial lectin-like oxidised LDL receptor 1 (LOX-1). This upregulation of LOX-1 has been hypothesised to occur in response to an excess accumulation of the highly reactive lipid peroxidation compound MDA, possibly due to the displacement of antioxidant enzyme PON-1 from within the HDL particle. These changes in turn result in an increased activation of the eNOS-inhibitor endothelial PKCβII, ultimately leading to a reduced capacity for endothelial NO generation [71]. Inactivation of PON-1 in healthy subjects in vitro has been shown to result in decreased eNOS phosphorylation at serine residue 1177 (an eNOS activating site), increased phosphorylation at threonine residue 495 (a deactivating site), and a subsequent decrease in NO bioavailability. This relationship may be mediated at least in part by the oxidative transformation of PON-1 by increased levels of pro-inflammatory enzymes such as MPO [72], as they have been shown to display reciprocal relationships. Furthermore, MPO and other oxidative products within HDL may directly bind to and activate endothelial cells, thereby further reducing NO production via eNOS coupling [73, 74].

### Inflammation Impairs HDL’s Antioxidant and Anti-inflammatory Capacity

The first evidence underpinning the concept of HDL ‘dysfunction’ came from the finding that HDL isolated from rabbits undergoing an acute phase response was unable to prevent lipid peroxidation or MCP-1 expression [47]. Since then, multiple other pro-inflammatory and pro-oxidant properties have been attributed to dysfunctional HDL particles; including an increased capacity to stimulate NADPH oxidase activity [44], an increased capacity for endothelial superoxide production [42, 69], an increased expression of cell adhesion markers such as VCAM-1 [43], and increased eNOS uncoupling [73]. Much of these functional changes are likely closely linked to those previously described when addressing reductions in NO bioavailability, with the replacement of protective proteins such as ApoA-I and PON-1 with atypical components such as MDA and MPO of particular importance. For example, while in vitro work has highlighted the ability of ApoA-I to remove seeding molecules responsible for LDL oxidation [40], this ability is lost in HDL isolated from patients with established CHD [75]. PON-1 is frequently observed to be reduced in inflammatory conditions and chronic disease [42, 47, 69], and genetic-knockout mice lacking PON-1 have been shown to have both impaired antioxidant effects and increased risk of atherosclerosis [38].

## HDL Function in Clinical Populations: Implications for CVD Risk

### Cardiovascular Disease

The cardiovascular disease process has been shown to exert widespread changes in HDL structure and function which may accelerate, rather than reduce, future risk of major adverse events. In a series of studies involving the LURIC, 4S, and KORA cohorts, the association between HDL-c and CVD mortality was assessed in almost 9000 patients undergoing coronary angiography who were stratified by median levels of serum SAA and SDMA [76, 77]. Remarkably, while patients with below median levels of both acute phase proteins showed the traditional inverse relationship between HDL-c and CVD outcomes, those with above median levels were observed to have the complete opposite response—i.e. a significantly increased risk of CVD mortality as HDL-c levels increased. Similar findings have also been reported when stratifying risk by levels of C-reactive protein (CRP) in the PREVEND study [78], and ApoC-III in two prospective case-control studies analysed within the Nurses Health and Health Professionals Follow-Up Studies [79], suggesting that stratification by inflammatory burden in CVD may help to identify the independent effects of ‘biologically effective’ and ‘biologically ineffective’ HDL particles. When assessing HDL function directly, cholesterol efflux capacity has been shown to be decreased in patients with CHD [80], while numerous endothelial protective functions appear to be impaired. Reduced NO bioavailability is evident in HDL isolated from patients with both established coronary heart disease and acute coronary syndrome [71]. Furthermore, and in contrast to its usual antioxidant and anti-inflammatory functions, HDL isolated from patients with CHD has also been shown to promote rather than protect against LDL oxidation [47, 75], as well as increasing the expression of adhesion markers such as MCP-1 [47], and promoting endothelial superoxide production in the presence of TNF-α [71].

### Type 2 Diabetes

HDL isolated from patients with type 2 diabetes demonstrates many of the structural and functional alterations previously described for CVD. Structural changes include increased SAA levels due to underlying low-levels of chronic inflammation [81], increased triglyceride content due to insulin resistance and elevated CETP activity [82], decreased ApoA-I and S1P due to displacement by other proteins [83], and increased oxidised lipids and advanced glycation end products (AGE) due to increased levels of systemic oxidative and glycaemic stress [84]. Cholesterol efflux is reduced [85]—possibly due to an impairment of both ABCA1 and SR-BI mediated pathways through oxidation and AGE—and LCAT activity is impaired [86]. Widespread impairments in the endothelial protective effects of HDL are also evident, as demonstrated by a recent study in which HDL isolated from patient with type 2 diabetes demonstrated impaired NO bioavailabilty, increased MPO and NADPH oxidase activity, increased superoxide production, and increased lipid peroxidation [44].

### Chronic Kidney Disease

The presence of reduced renal function in chronic kidney disease (CKD) leads to an accumulation of acute phase proteins within a circulating uremic milieu, many of which are capable of negatively altering HDL structure and function. HDL isolated from patients on haemodialysis is enriched with numerous components detrimental to HDL function (e.g. SAA, ApoC-III, A1AT, and triglycerides), and has been observed to have a reduced capacity to initiate RCT from macrophages [87]. Of particular importance in CKD is a significant increase in the acute phase protein symmetric dimethylarginine (SDMA), whose incorporation into the HDL particle is associated with impairments in HDL-mediated RCT and endothelial NO production in a dose-dependent manner [43, 70, 77]. This effect becomes progressively pronounced as kidney function declines, as demonstrated by a 40–60% decline in HDL-mediated endothelial NO bioavailability in patients with stage 5 CKD as opposed to a 20% increase in healthy controls [43]. Interestingly, these changes are largely reversed following kidney transplantation in children, but not adults—suggesting that HDL’s vascular protective functions may be at least partially restored in the young [43]. Using genetic knockout mice models to investigate the pathways underlying this relationship, Speer and colleagues identified toll-like receptor 2 (TLR2) as the likely signalling receptor responsible, suggesting that structural modifications to HDL caused by CKD may result in damage-associated molecular patterns within the HDL particle that trigger a chronic innate immune response [74]. Further post-translational modifications to other circulating proteins may also affect function. Albumin isolated from haemodialysis patients is heavily oxidised and markedly impairs cholesterol efflux in vitro; likely due to its high-affinity for SR-BI reducing binding sites for HDL-mediated RCT [88].

### Autoimmune Diseases

HDL dysfunction has recently been demonstrated in a range of autoimmune diseases, including type 1 diabetes [89], rheumatoid arthritis [90, 91], systemic lupus erythematosus [92], and primary antiphospholipid syndrome [69]. Despite being at increased risk of dyslipidaemia, patients with type 1 diabetes commonly demonstrate elevated—rather than reduced—levels of HDL-c. Multiple studies have reported the presence of multiple dysfunctional properties to these particles, however, including reduced cholesterol efflux capacity [93, 94], reduced PON-1 activity [95], increased MPO activity [96], and decreased levels of S1P [97]. These modifications appear to be independent of HbA1c [93], and may at least partially explain the increased risk of CVD in patients with the highest HDL-c levels [98]. HDL isolated from patients with rheumatoid arthritis, systemic lupus erythematosus, and antiphospholipid syndrome also display widespread structural changes, resulting in a pro-inflammatory phenotype that worsens as systemic inflammatory burden increases [69, 91]. Treatment with anti-inflammatory medications in these patients may partially reverse these structural and functional modifications, as evidenced by decreases in SAA and LBP content, increased NO bioavailability and PON-1 activity, and decreased SO production in individuals with rheumatoid arthritis treated with the monoclonal antibodies infliximab and adalimumab [99, 100].

### Periodontitis

Periodontal disease represents one of the most prevalent chronic inflammatory conditions in the world today, affecting approximately 20–50% of the global population [101]. Numerous vasoprotective functions of HDL have been shown to be impaired in periodontal patients compared to age-matched controls, including impaired endothelial NO bioavailability, increased superoxide production, and reduced PON-1 activity [42]. When using an intensive periodontal treatment intervention as an experimental model of acute inflammation, further transient decreases in both endothelial NO availability and PON-1 activity—but not RCT—are apparent in the following 24 h, highlighting the vulnerability of HDL to rapidly remodel when exposed to an inflammatory response. These changes were accompanied by substantial increases in acute phase reactants such as SAA, C3, and prothrombin, and returned to baseline levels following resolution of the inflammatory response.

## Where Next for HDL as a Therapeutic Target?

Given recent advances in our understanding of HDL’s myriad functions, a limited number of population studies in recent years have attempted to link individual markers of HDL function, rather than HDL-c levels, to future risk of CVD. The largest and most rigorous of these have used cholesterol efflux capacity as the functional measure of interest, and have found increased efflux to be associated with a reduced risk for CVD in some [102–104], but not all [23], of these studies. In the largest of these to date, cholesterol efflux capacity was found to be inversely related to incidence of major CV events in nearly 3000 adults free from CVD at baseline and followed-up for over 9 years as part of the Dallas Heart Study (hazard ratio 0.33 [0.19–0.55] for top quartile of efflux compared to bottom) [102]. Interestingly, while HDL-c levels were found to be inversely associated with well-established CV risk factors such as obesity or insulin resistance, cholesterol efflux capacity was not, suggesting that changes in RCT may occur through distinct mechanisms to those affecting overall HDL-c levels. These findings were supported in a more recent nested case-control analysis in 3500 middle-aged to elderly individuals participating in the EPIC-Norfolk study, where increased cholesterol efflux was once again associated with a substantially reduced risk of incident CV events (fully adjusted odds ratio per SD increase in efflux capacity=0.80 [0.70–0.90] for those in the top tertile of cholesterol efflux capacity compared to those in the bottom) [103].

Alongside hard clinical endpoints, further encouraging findings relating changes in function to surrogate measures of CVD risk have also been reported in a number of other studies. Using carotid intima-media thickness (cIMT) and angiographically confirmed presence of CHD as surrogate markers of atherosclerotic disease progression, Khera and colleagues showed a substantially decreased presence of coronary disease in individuals within the top quartile for cholesterol efflux (OR=0.74 [0.61–0.89]) [105]. In children with CKD, Shroff et al. reported an inverse relationship between HDL-mediated NO bioavailability and multiple surrogate markers of arterial health, including cIMT and pulse-wave velocity (a measure of arterial stiffness) [43]. Likewise, in a case-control study of women with and without the autoimmune condition primary antiphospholipid syndrome, these same surrogate measures were found to be inversely related to PON-1 activity, and were accompanied by additional detrimental changes in HDL functionality such as decreased NO bioavailability and increased SO production [69]. These results suggest that interventions to modify HDL function, rather than simply increasing HDL-c levels—may still hold therapeutic potential for reducing atherosclerotic risk.

Efforts to translate these into clinical benefits in randomised trials, however, have once again proven to be challenging. For example, although 3 months of niacin therapy in patients with T2D was shown to significantly improve both HDL-c levels and numerous measures of HDL function [44], the use of this same drug in individuals at high risk for CVD displayed no clinical benefit in either the AIM-HIGH and HPS2-THRIVE trials [6, 7]. Similar issues were encountered in studies assessing the efficacy of recombinant ApoA-I infusions as a therapeutic target, which have been proposed to reduce atherosclerotic risk via their promotion of ABCA1-mediated reverse cholesterol transport. While findings from both animal studies [106] and a small-scale clinical trial in humans [107] suggested evidence of atherosclerotic plaque regression, the recent publication of two large-scale randomised controlled trials utilising both the wild-type and mutated versions of ApoA-I (the latter termed ApoA-I Milano and considered to be particularly beneficial) have once again found no clinical benefit, despite increases in cholesterol efflux capacity of up to 90% [108, 109].

It is clear that if modification of HDL function is to be pursued as a therapeutic target, further experimental studies are required to identify the most relevant pathways for intervention, and genetic studies are required to provide a causal link between these pathways and CVD. Once appropriate pathways are identified, standardisation of high-throughput functional assays for use in routine clinical practice will also be essential, as this will permit relationships between HDL function and CVD risk to be tested in large-scale cohorts and trials, and will allow comparisons of results between studies. Of note, all major studies investigating cholesterol efflux to-date have used different techniques to quantify efflux capacity, an observation which may potentially explain discrepancies in findings reported so far. Most importantly though, lessons must be learned from previous failings involving HDL-c, where the presence of observational correlations were long-considered to represent a causal relationship between HDL levels and CVD risk. Despite some promising results relating a number of markers of HDL function to future risk of disease, successful completion of large-scale randomised clinical trials altering specific functional properties of HDL are needed to support the use of HDL function as a therapeutic target.

## Conclusions

Despite HDL-c’s well-established inverse relationship with CVD risk, it does not play a causal role in CVD risk reduction, and is therefore an unsuitable target for therapeutic intervention. While our understanding of the mechanisms underlying HDL function and dysfunction have been significantly enhanced in recent years, the wide range of different functions elicited by HDL, their vulnerability to modification by external inflammatory responses, and a lack of standardised assays for the measurement in routine clinical practice makes the translation of these findings challenging. While research is continuing to tease out the complexities of this highly heterogenous particle, potential diagnostic and therapeutic strategies involving the modification of HDL remain elusive.

## Abbreviations

A1AT: Alpha-1-antitrypsin
ABCA1: ATP binding cassette transporter A1
ABCG1: ATP binding cassette transporter G1
AGE: Advanced glycation end products
ApoA-I: Apolipoprotein A1
ApoA-II: Apolipoprotein A2
ApoC-III: Apolipoprotein C3
C3: Complement component 3
CETP: Cholesterol ester transfer protein
CHD: Coronary heart disease
cIMT: Carotid intima-media thickness
CKD: Chronic kidney disease
CRP: C-reactive protein
CVD: Cardiovascular disease
cIMT: Carotid intima-media thickness
CVD: Cardiovascular disease
eNOS: Endothelial nitric oxide synthase
GWAS: Genome-wide association study
HbA1c: Glycated haemoglobin
HDL: High-density lipoprotein
HDL-c: High-density lipoprotein cholesterol
ICAM-1: Intercellular adhesion molecule 1
LBP: Lipopolysaccharide binding protein
LDL: Low-density lipoprotein
LCAT: Lecithin-cholesterol acyltransferase
LOX-1: Lectin-like oxidised LDL receptor 1
Lp-PLA_2_: Lipoprotein-associated phospholipid A2
LSF: Lysosulfatide
MAPK: Mitogen-activated protein kinase
MCP-1: Monocyte chemoattract protein 1
MDA: Malondialdehyde
MPO: Myeloperoxidase
NADPH: Nicotinamide adenine dinucleotide phosphate
NO: Nitric oxide
oxLDL: Oxidised LDL
PI3K: Phosphatidylinositol 3-kinase
PLTP: Phospholipid transfer protein
PON-1: Paraoxonase 1
RCT: Reverse cholesterol transport
RNA: Ribonucleic acid
ROS: Reactive oxygen species
S1P: Sphingosine-1-phosphate
S1P3: Sphingosine-1-phosphate receptor 3
SAA: Serum amyloid A
SDMA: Symmetric dimethylarginine
SPC: Sphingosylphosphorylcholine
SR-BI: Scavenger receptor class B type I
SO: Superoxide
T2D: Type 2 diabetes
TLR2: Toll-like receptor 2
TNFα: Tumour necrosis factor alpha
VCAM-1: Vascular cellular adhesion molecule 1
VLDL: Very low density lipoprotein
